# The Erie County Eye, Ear, and Throat Infirmary

**Published:** 1891-01

**Authors:** 


The Erie County Eye, Ear, and Throat Infirmary.—There have been some recent changes in the attending staff of this institution. Dr. F. Whitehill Hinkel has resigned, and Dr. Frank H. Potter has been appointed Surgeon-in-charge of the Nose and Throat Department. Dr. Hinkel retains his membership upon the Board of Trustees, and it is expected that in the near future he will resume a position upon the active staff. This Infirmary, as its name indicates, limits its treatment to patients suffering from diseases of the eye, ear, nose, and throat. It is centrally located, at the corner of Huron and Main streets, and is open daily for the reception of patients. Its records show that during the last six months it has treated over four hundred patients. The profession generally, not only of the city, but of the county — in the surrounding towns, and in the country districts — may be assured that worthy patients, unable to pay for medical services, and requiring treatment in the various departments represented in this Infirmary, will receive careful and conscientious attention.

